# Transcriptional and Mutational Profiling of B-Other Acute Lymphoblastic Leukemia for Improved Diagnostics

**DOI:** 10.3390/cancers13225653

**Published:** 2021-11-12

**Authors:** Philippe Chouvarine, Željko Antić, Jana Lentes, Charlotte Schröder, Julia Alten, Monika Brüggemann, Enrique Carrillo-de Santa Pau, Thomas Illig, Teresa Laguna, Denis Schewe, Martin Stanulla, Ming Tang, Martin Zimmermann, Martin Schrappe, Brigitte Schlegelberger, Gunnar Cario, Anke K. Bergmann

**Affiliations:** 1 Institute of Human Genetics, Hannover Medical School (MHH), 30625 Hannover, Germany; chouvarine.philippe@mh-hannover.de (P.C.); antic.zeljko@mh-hannover.de (Ž.A.); lentes.jana@mh-hannover.de (J.L.); schroeder.charlotte@mh-hannover.de (C.S.); illig.thomas@mh-hannover.de (T.I.); tang.michelle@mh-hannover.de (M.T.); schlegelberger.brigitte@mh-hannover.de (B.S.); 2 Berlin-Frankfurt-Münster ALL Study Group Germany (BFM-G), Department of Pediatrics, University Medical Center Schleswig-Holstein, Campus Kiel, 24105 Kiel, Germany; julia.alten@uksh.de (J.A.); denis.schewe@uksh.de (D.S.); 3 Department of Hematology, University Hospital Schleswig-Holstein, Campus Kiel, 24105 Kiel, Germany; m.brueggemann@med2.uni-kiel.de (M.B.); m.schrappe@pediatrics.uni-kiel.de (M.S.); gunnar.cario@uksh.de (G.C.); 4 Computational Biology Group, Precision Nutrition and Cancer Research Program, IMDEA Food Institute, 28049 Madrid, Spain; enrique.carrillo@imdea.org (E.C.-d.S.P.); teresa.laguna@imdea.org (T.L.); 5 Pediatric Hematology and Oncology, Hannover Medical School (MHH), 30625 Hannover, Germany; stanulla.martin@mh-hannover.de (M.S.); zimmermann.martin@mh-hannover.de (M.Z.)

**Keywords:** whole-transcriptome sequencing, B-cell precursor acute lymphoblastic leukemia, gene fusions, treatment stratification biomarkers

## Abstract

**Simple Summary:**

Pediatric acute lymphoblastic leukemia is the most common malignancy in children. Based on the genetic characteristics of the tumor, patients are risk-stratified and treated with different treatment intensities. However, in a proportion of cases, known as B-other, no genetic alterations relevant for risk stratification are found with routine diagnostic procedures. In this study, we performed RNA sequencing, a comprehensive and cutting-edge method, of 185 children with B-other leukemia and analyzed gene fusions, expression profiles and mutations. Furthermore, we validated our findings using commonly used diagnostic techniques. Our results identified a subgroup of cases clustering with known leukemia subtypes, e.g., *DUX4*-positive, and subgroups characterized by mutations in *PAX5* and *IKZF1*, resulting in more cases being assigned to a defined subgroup. Moreover, we identified new fusion partners of *ETV6*, *IKZF1* and *PAX5*. Our data demonstrate the applicability and technical considerations for the use of RNA sequencing to personalize genetic diagnostics in pediatric leukemia.

**Abstract:**

B-cell precursor acute lymphoblastic leukemia (BCP-ALL) is the most common cancer in children, and significant progress has been made in diagnostics and the treatment of this disease based on the subtypes of BCP-ALL. However, in a large proportion of cases (B-other), recurrent BCP-ALL-associated genomic alterations remain unidentifiable by current diagnostic procedures. In this study, we performed RNA sequencing and analyzed gene fusions, expression profiles, and mutations in diagnostic samples of 185 children with BCP-ALL. Gene expression clustering showed that a subset of B-other samples partially clusters with some of the known subgroups, particularly *DUX4*-positive. Mutation analysis coupled with gene expression profiling revealed the presence of distinctive BCP-ALL subgroups, characterized by the presence of mutations in known ALL driver genes, e.g., *PAX5* and *IKZF1*. Moreover, we identified novel fusion partners of lymphoid lineage transcriptional factors *ETV6*, *IKZF1* and *PAX5*. In addition, we report on low blast count detection thresholds and show that the use of EDTA tubes for sample collection does not have adverse effects on sequencing and downstream analysis. Taken together, our findings demonstrate the applicability of whole-transcriptome sequencing for personalized diagnostics in pediatric ALL, including tentative classification of the B-other cases that are difficult to diagnose using conventional methods.

## 1. Introduction

B-cell precursor acute lymphoblastic leukemia (BCP-ALL) represents the most common childhood malignancy [1,2,3,4]. Improvements in the treatment strategies and the introduction of new drugs have resulted in a dramatic increase in the overall survival of children with BCP-ALL, reaching around 90% in contemporary treatment protocols [5,6,7]. The treatment of BCP-ALL is based on the identification of genetic and clinical markers in order to risk stratify children with the disease into different treatment arms. The goal of this approach is to provide more intensive treatment for patients at risk of developing a relapse, e.g., *BCR-ABL1*-positive or *KMT2A* rearranged, while limiting toxic effects for patients with favorable prognoses, e.g., *ETV6-RUNX1*-positive or hyperdiploid [8,9,10]. Historically, in a relevant proportion of BCP-ALL, no major genetic alteration could be detected with conventional cytogenetic approaches, and these cases were referred to as B-other subtype [9,11,12]. With the advent of the high-throughput and next-generation sequencing (NGS) techniques, new leukemia-driving alterations emerged from the B-other subgroup, enabling their further risk-based stratification. For example, the identification of the *BCR-ABL1*-like (Ph-like) BCP-ALL was made based on the expression signature in the subset of B-other cases, which was similar to the one observed in *BCR-ABL1*-positive BCP-ALL, and shared unfavorable prognoses [13,14]. Later studies revealed that this group is characterized by heterogeneous genomic alterations but also includes cases with good outcomes, despite the *BCR-ABL1*-like expression signature [15,16]. This enabled further treatment optimization and identification of cases that may benefit from targeted therapies, e.g., tyrosine kinase inhibitors (TKI), imatinib and dasatinib, in cases with *ABL1* class fusions [17,18]. In the subsequent studies, the heterogeneous B-other subgroup was further dissected, resulting in the discovery of new leukemia driving alterations, e.g., *MEF2D*, *ZNF384* and *NUTM1* rearranged [19,20,21,22,23], as well as heterogeneous subgroups with expression profiles similar to classic BCP-ALL subtypes, e.g., *ETV6-RUNX1*-like [24]. In addition, other alterations emerged as prognostically relevant, e.g., *IKZF1* deletions resulting in a further personalization of the standard treatment protocols [14,25,26]. However, despite this remarkable progress, BCP-ALL remains one of the leading causes of mortality in childhood [27]. Furthermore, in the contemporary protocols, chemotherapy intensity was raised to the maximal levels of tolerability, and further improvements in the outcome will depend on the characterization and re-classification of cases in the B-other subgroup, as well as the development of new drugs targeting frequently altered pathways in the BCP-ALL. 

In order to further deconvolute the heterogeneous B-other subtype and show the applicability of whole-transcriptome sequencing for the routine diagnostics and personalized medicine in pediatric ALL, we performed whole-transcriptome sequencing of 185 BCP-ALL cases at diagnosis, treated in contemporary AIEOP-BFM ALL trials. We subsequently analyzed their fusion transcripts, expression profiles and mutational landscapes and validated our findings using karyotyping, FISH, immunophenotyping, arayCGH and Sanger sequencing. In addition, we tested the applicability of RNA-sequencing (RNA-seq) in cases with a low blast count and the use of EDTA tubes for sample collection instead of PAXgene RNA stabilizing tubes.

## 2. Materials and Methods

### 2.1. Patient Cohort, Sample Preparation, DNA and RNA Extraction

In this study, we included 185 patients diagnosed with pediatric BCP-ALL, of which 174 patients were treated according to the Associazione Italiana Ematologia Oncologia Pediatrica (AIEOP)-Berlin-Frankfurt-Münster (BFM) ALL 2009 (*n* = 168) or Interfant 06 (*n* = 6) treatment protocols, in the differential gene expression analysis, mutation calling and gene fusion analysis. Furthermore, 12 samples from 6 patients treated according to the AIEOP-BFM ALL 2017 protocol were included for comparison between PAXgene RNA stabilizing and tubes containing EDTA. Finally, in order to test whether samples with a low blast count can be used to detect the fusion transcript, we included samples from five cases diagnosed with pediatric ALL and low blast count (range: 48–7%). The usage of leukemic samples was approved by the institutional review board of the Medical Faculty of the Christian-Albrechts-University Kiel (BFM-ALL 2000: B257/01; AIEOP-BFM ALL 2009: A177/09; INTERFANT 06: A103/08, AIEOP-BFM ALL 2017: A105/18).

According to the above-mentioned study protocols, each sample was tested for the presence of *BCR-ABL1*, *ETV6-RUNX1*, rearrangements involving the *KMT2A* gene, hyperdiploidy and hypodiploidy. The absence of these risk-stratifying rearrangements was the prerequisite for inclusion into the study. The median tumor cell content was 93.5% (range: 41–98.5%). Patient characteristics are summarized in Table S1.

DNA and RNA were isolated from mononuclear cells obtained from bone marrow (BM) aspirate or peripheral blood (PB) at the time of diagnosis and viably frozen in the DMSO. The isolation of DNA from EDTA BM was performed using the QIAamp DNA Blood Midi Kit (Qiagen, Hilden, Germany), while RNA was isolated using the RNeasy Kit (Qiagen, Hilden, Germany). For each of the six patients, matched PAXgene and EDTA samples were obtained from the same bone marrow aspirate, and RNA was isolated within 24 h from the bone marrow sampling. The concentration of the isolated RNA was measured using Qubit, while the quality assessment was performed with a bioanalyzer using the RNA 6000 Nano kit. 

### 2.2. Whole-Transcriptome RNA Sequencing

Whole-transcriptome RNA sequencing was performed using the TruSeq Stranded Total RNA Library Prep kit (Illumina, San Diego, CA, USA) according to the manufacturer’s instructions using 200 ng of RNA, with an RIN score ≥9. Sequencing was done on an Illumina NovaSeq sequencer using S4 Flow Cell. The average number of read pairs per sample was 121.3 million (median ± standard deviation (SD): 118.6 ± 25.9 million).

### 2.3. Karyotyping, FISH Analysis and Array CGH

For selected samples, fusions detected via RNA sequencing were validated using karyotyping, FISH analysis on metaphase and interphase nuclei and ArrayCGH analysis (Table S2). Karyotyping, fluorescence R-banding and FISH were performed as previously described [28]. In brief, short-term cultures (24–48 h) were set up from viably frozen cells. For karyotyping and fluorescence R-banding, cells in metaphases were harvested and fixed using the HANABI P2Plus Metaphase Chromosome Harvester (ADS Biotec, Omaha, NE, USA). Cell suspension was added on microscope slides, dried and stained using Chromomycin A3 (Merck, Darmstadt, Germany) for 45 min at 4 degrees Celsius, following counterstaining using Methyl Green (Sigma-Aldrich, Burlington, MA, USA) for five minutes. Chromosome analysis was performed using Axioplan 2 imaging and Axio Imager.Z2 microscopes (Zeiss, Jena, Germany). For FISH analysis, cells were fixed on microscope slides with cold methanol (Chemsolute, Renningen, Germany) for five minutes, following three minutes of digestion with pepsin (Sigma-Aldrich, Burlington, MA, USA) and 10 min of fixation with formalin (Carl Roth, Karlsruhe, Germany). FISH probes were mixed with hybridization buffer and added to fixed nuclei, following denaturation for 10 min at 80 degrees Celsius. FISH analysis was done using the following probes: Vysis ETV6 Break Apart FISH, Vysis LSI BCL2 Break Apart FISH, Vysis LSI MLL Dual Color, Break Apart Rearrangement Probe (ASR) (Abbott, Wiesbaden, Germany), IGH Plus Breakapart, E2A (TCF3) Break Apart, EPOR Breakapart, PAX5 Breakapart, CRLF2 Breakapart, ABL1 Breakapart, PDGFRB Breakapart (Cytocell, Cambridge, UK) [29]. Upon overnight incubation at 37 degrees Celsius in a humidified dark chamber, slides were washed and counterstained for 10 min with DAPI (Sigma-Aldrich, Burlington, MA, USA). The evaluation of signals was carried out using an Axioskop 2 plus microscope (Zeiss, Jena, Germany).

ArrayCGH analysis was performed by hybridizing 500 ng of patient DNA using a 400K SurePrint G3 Custom CGH Human Genome Microarray (e-Array design 84704, Agilent Technologies, Waldbronn, Germany) according to the manufacturer’s instructions.

### 2.4. Immunophenotyping

Immunophenotyping was done using the diagnostic standards developed and approved by the AIEOP-BFM ALL FLOW-Study Group, which are based on the WHO 2016 criteria, the EGIL classification and the Bethesda 2006 recommendations [30,31]. To validate the *DUX4*-positive subtype, the expression of CD371 (CLL1) was determined with an 8-color combination of antibodies against CD45, CD19, CD34, CD2, CD10, CD14, CD33 and CD371 at diagnosis [32].

### 2.5. Fusion Detection

The sequencing data were processed using the megSAP RNA-seq pipeline (https://github.com/imgag/megSAP; accessed on 1 May 2019), which includes pre-processing (QC and adapter trimming), aligning to the GRCh38 genome reference and annotation. Fusions were detected using megSAP (release 0.2) and Arriba (version 2.0.0) [33] and filtered based on the presence of the fusion partners in a “white list” of ALL-relevant genes obtained from the literature (Table S3). Other fusions were only kept if they had more than 10 supporting reads (i.e., junction or spanning reads). Furthermore, we kept only known fusions from the Mitelman Database of Chromosome Aberrations and Gene Fusions in Cancer. Fusions were further prioritized based on the relevant literature [11,19,34]. 

### 2.6. Mutation Analysis

Single base substitutions (SBS) and indels were identified using the Mutect2 of GATK (version 4.1.8.1) [35], with modified settings for RNA-seq data (using the SplitNCigarReads utility and adding-read-filter NotSupplementaryAlignmentReadFilter and --disable-read-filter ReadStrandFilter as flags for the FilterMutectCalls utility). Somatic variant calling by Mutect2 was performed using a panel of normals from the GATK resource bundle. Somatic variants were further processed by the Mutect2 filter (FilterMutectCalls utility), and additional filters were applied to remove variants with (1) less than six alternative allele reads; (2) allele frequency in tumor (as determined by Mutect2) less or equal 0.1; (3) co-occurrence in at least 40% of the samples; (4) indels positioned within 20 bp of each other. Furthermore, we removed variants located in introns, IGR, 5′ flanking, splice site as well as silent variants upon annotation with Funcotator (GATK). A separate dataset was created, in which silent variants were retained, for further analyses using MAFTools [36]. Variants were further annotated using VEP (release 103), and we filtered out the variants that had an allele frequency >0.01% (>1 in 10,000) in at least one ethnic group in 1000 Genomes, ExAC or GnomAD datasets, a CADD PHRED score <15 or were classified as benign, tolerated or neutral (PolyPhen-2, SIFT and Condel, respectively). Furthermore, we only kept variants rated to have high or moderate impact (i.e., “transcript_ablation”, “splice_acceptor_variant”, “splice_donor_variant”, “stop_gained”, “frameshift_variant”, “stop_lost”, “start_lost”, “transcript_amplification”, “inframe_insertion”, “inframe_deletion”, “missense_variant”, and “protein_altering_variant”). In the second set that was created for the MAFTools analyses, only variants that had an allele frequency >0.01% in at least one of the ethnic groups (GnomAD) were removed. The remaining variants are reported for each BCP-ALL subgroup in Table S4. We used MAFTools to query the mutational composition of the BCP-ALL subgroups based on: (i) variant classification (missense, nonstop, nonsense); (ii) variant type (SNP, DNP, TNP, etc.); (iii) single base substitution type (T > C, C > T, etc.); (iv) top genes with the highest number of mutations co-occurring in the highest number of samples; (v) and co-occurrence of genes with mutations.

### 2.7. Gene Expression Profiling

To perform gene expression profiling, we first generated read alignments translated into transcript coordinates using the STAR aligner (version 2.7.0f) [37], which is integrated into our slightly modified version of megSAP (the modification was limited to the addition of the quantMode TranscriptomeSAM parameter to the STAR command line). Gene-level quantification was performed using RSEM (version 1.3.0) [38]. The heatmaps are based on variance stabilized transformed expression values generated using the DESeq2 R package [39].

### 2.8. Sample Classification

Upon mutation calling and gene expression profiling, we classified the samples based on the presence of fusions and mutations previously known from the literature [11,19,34]. Further, we used karyotyping, FISH, ArrayCGH, Sanger sequencing and flow cytometry data to validate assigned groups. The resulting sample annotation was further validated by dimensionality reduction techniques for data visualization: t-Distributed Stochastic Neighbor Embedding [40] (t-SNE; R package Rtsne, version 0.15) and Uniform Manifold Approximation and Projection [41] (UMAP; R package umap, version 0.2.7.0). The Rtsne function was used with the following parameters set: perplexity = 20 and max_iter = 50000. The umap function was used with default parameters. The input for these algorithms was generated by within-lane GC normalization (R package EDASeq [42]) and variance stabilization (vst function in DESeq2 [39]) of the expression count data generated by RSEM. T-SNE and UMAP sample clustering was performed on 650 of the most variable differentially expressed genes (DEGs) that were identified using median absolute deviation (R mad function) of the variance stabilized expression data. The most variable DEGs were selected from the union of DEGs (FDR < 0.05) from comparisons of each subgroup with at least three samples vs. all other samples in the study. We have tried a range of the most variable DEGs from 300 to 3000 in increments of 50, and the set with the top 650 DEGs produced the best separation of all subgroups in both t-SNE and UMAP clustering. 

### 2.9. Differential Gene Expression

For differential gene expression analysis, we used the EDASeq/DESeq2 pipeline mentioned above. The identified subgroups were compared against all the other samples combined. Additionally, we performed GO and pathway overrepresentation analysis of the identified DEGs using the online tool Erichr [43,44,45].

To compare gene expression profiles of the samples collected using PAXgene RNA stabilizing and tubes containing EDTA from the same patients, we ran DESeq2 with the design setting applicable for paired data (design = ~patient + condition). The resulting differential expression data with a q-value < 0.05 (i.e., *p*-value adjusted for false discovery rate (FDR) using the Benjamini–Hochberg procedure) were considered significant. To determine which genes and respective pathways are affected most by the different sample collection tubes (PAXgene vs. EDTA), we created three subgroups of DEGs based on the effect size cutoff formulated as the absolute value of the log-normalized fold change: |Log2(fold change)| >1 (fold change >2 or <0.5), |Log2(fold change)| > 2 (fold change >4 or <0.25) and |Log2(fold change)| > 3 (fold change >8 or <0.125). The resulting gene sets were analyzed using the pre-ranked analysis option in GSEA with the default gene number range (15–500) [46,47]. The ranking was based on the expression score calculated as Log2(fold change) × (−Log10(q-value)). GSEA was run on the complete hallmark gene set collection (h.all.v7.2.symbols.gmt). The enriched gene sets with the FDR-adjusted *p*-value < 0.05 are reported in this manuscript.

### 2.10. Differential Splicing Analysis

To perform differential splicing analysis, we first generated read alignments translated into transcript coordinates using the STAR aligner (--quantMode TranscriptomeSAM), which is integrated into our slightly modified version of megSAP. RSEM was used to quantify the isoform-level expression data. Differential splicing analysis was performed using NBSplice (version 3.13) [48] for the analysis of the isoform-level output from RSEM.

## 3. Results

A total of 174 cases (102 male and 72 female) with BCP-ALL and treated according to AIEOP-BFM ALL 2009 (*n* = 168) or Interfant 06 (*n* = 6) treatment protocols were included in this study. The median age at diagnosis was 7 years (range: 0.2–18.6; Table S1). In addition, 12 samples from six patients treated according to the AIEOP-BFM ALL 2017 treatment protocol were included for comparison between PAXgene RNA stabilizing and tubes containing EDTA (Table S1). Furthermore, five samples from the patients with low blast counts were used to examine the applicability of whole-transcriptome analysis to detect fusion transcripts in samples with low blast counts. Following the RNA sequencing of samples obtained at diagnosis, we performed analysis and filtering of the detected fusion transcripts, which resulted in the identification of 203 fusions known to frequently occur in BCP-ALL (distribution by subgroup is shown in Table S5; for complete fusion detection output, see File S1). These 203 fusions were used for sample classification (prior to manual review) based on the highest percent of supporting reads. The most frequently identified gene fusion was *P2RY8-CRLF2* (*n* = 19), followed by the fusions involving *ZNF384* (*n* = 10), *PAX5* (*n* = 10), *ETV6* (*n* = 5), *NUTM1* (*n* = 4), *EBF1-PDGFRB* (*n* = 4), *MEF2D-BCL9* (*n* = 3) and *IGH-EPOR* (*n* = 2). To our surprise, we also identified six cases with cryptic *KMT2A* rearrangements, which were not detected by FISH analysis. These *KMT2A* rearrangements involved two cases of each *MLLT1*, *MLLT3* and *USP2* genes as fusion partners. Other identified gene fusions were non-recurring. Furthermore, we have identified 13 fusions involving lymphoid lineage transcriptional factors *ETV6*, *IKZF1* and *PAX5*, with fusion partners, which, to best of our knowledge, were previously not reported in pediatric ALL (Table S2). Mutation calling analysis identified 4108 predicted pathogenic variants (distribution by subgroup is shown in Table S4), including hotspot mutations in known ALL drivers *PAX5* and *IKZF1*. In order to confirm these findings (obtained after detection of fusion transcripts, mutation analysis, and gene expression analysis) and to further characterize samples that were not assigned to any of the known BCP-ALL subtypes, we performed additional validation using karyotyping, FISH, arrayCGH and immunophenotyping in distinct samples with the available material (Table S2). Upon the completion of all analysis and validation steps, we assigned the samples to the following BCP-ALL subgroups: *BCR-ABL1* (Ph)-like (*n* = 31), *DUX4*-positive (*n* = 16), *ETV6-RUNX1*-like (*n* = 3), *IKZF1* (p.N159Y) (*n* = 1), *KMT2A*-rearranged (*n* = 6), *MEF2D-BCL9* (*n* = 3), *NUTM1*-rearranged (*n* = 4), *PAX5* (p.P80R) (*n* = 6), *PAX5* alt (*n* = 6), *TCF3-HLF* (*n* = 1), *TCF3-PBX1* (*n* = 1), *ZNF384*-rearranged and like (*n* = 10) and iAMP21 (*n* = 3), while 84 samples remained unassigned at this point (Table S2). 

### 3.1. B-Other Samples Cluster with Known B-ALL Subgroups Based on Gene Expression 

**Figure 1:**
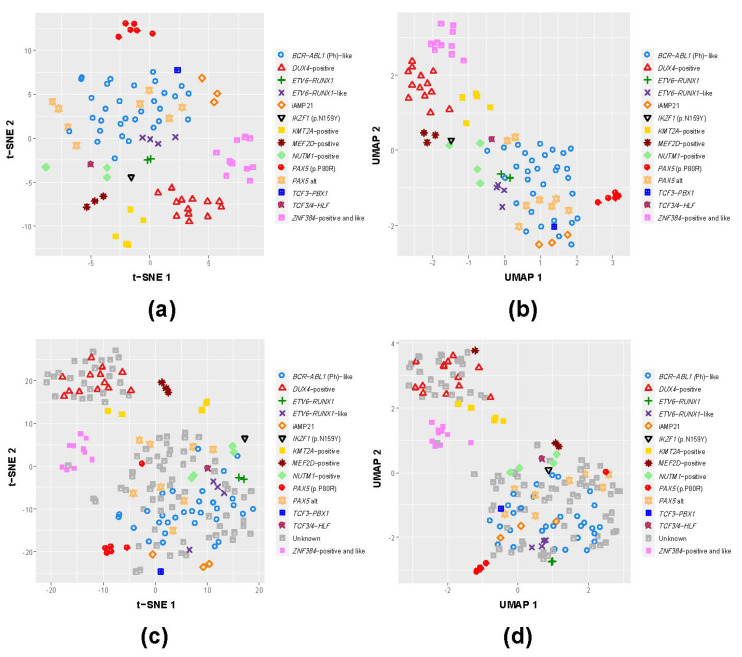
Whole-transcriptome expression data provides the means to cluster known BCP-ALL subgroups and tentatively classify co-clustering unknown samples. We used two dimensionality reduction visualization methods: t-Distributed Stochastic Neighbor Embedding (t-SNE; **a**,**c**) and Uniform Manifold Approximation and Projection (UMAP; **b**,**d**) for clustering the samples of the entire cohort, excluding the unknown samples (**a**,**b**) and including the unknown samples (**c**,**d**). Many unknown samples cluster with known subgroups, particularly *DUX4*, thereby providing the means for tentative classification of such unknown (B-other) samples.

Previous studies have demonstrated that a subset of B-other cases with heterogeneous genomic fusions tend to cluster with known subtypes, e.g., *BCR-ABL1*-like and *ETV6-RUNX1*-like. In order to examine the presence of these subgroups and classify the remaining unassigned samples, we selected the union of the top 650 DEGs (see Methods) and performed data dimensionality reduction using t-SNE and UMAP on the well-defined and validated BCP-ALL subtypes in our cohort (Figure 1a,b). Upon examining the distribution of the clusters formed by individual samples and confirming that these are represented by their respective genomic fusions, we performed the same analysis using both samples with the known BCP-ALL subtypes and a group of cases without known major genomic aberrations (Figure 1c,d). Cases with distinctive and confirmed BCP-ALL subtypes again clustered together, while for a subset of cases without known genomic alterations, we observed their co-clustering with these confirmed BCP-ALL subtypes, namely *BCR-ABL1* (*n* = 59), *ETV6-RUNX1* (*n* = 13) and *ZNF384*-rearranged (*n* = 1), representing a previously reported subgroup of cases with expression profiles similar to the mentioned subtypes. Notably, for the *DUX4*-positive subtypes (*n* = 13), we observed 27 cases without detectable genomic alterations clustering together, a finding which may be relevant for risk treatment stratification (Figure 1c,d). Of note, differential expression analysis of the *DUX4* group and the B-other subgroup clustering together with *DUX4*-positive cases showed that their differential expression profiles, though distinct, were still the closest compared to the profiles of the other subgroups. Particularly, the *DUX4* co-clustering B-other subgroup had 14 pathways significantly overrepresented by DEGs shared with the *DUX4* group, while the next closest subgroup was *BCR-ABL1* with 11 shared overrepresented pathways (File S2). In summary, through our approaches, we were able to assign 172 samples into one of the B-ALL subgroups, including the majority (82/84; 98%) of previously unassigned B-other cases. 

### 3.2. Splicing Profiles of the Known BCP-ALL Subgroups Provide Additional Information for Potential Improvement in Diagnostics of B-Other Samples

To further unravel differences between ALL subtypes, as well as B-other cases co-clustering with known BCP-ALL subtypes, we performed differential splicing analysis and identified isoforms that were differentially expressed in a particular ALL subtype compared to the rest of the cohort. Overall, we did not observe many significantly differentially expressed isoforms (FDR < 0.05) in the investigated subgroups, and the highest number of such isoforms was 11 for the cases with iAMP21. Nevertheless, differential splicing analysis proved to be useful for extending expression signatures of the known BCP-ALL subgroups and validation of the co-clustering of B-other sample subsets unassigned to any known subgroups. In particular, we performed differential splicing analysis on the subset of B-other cases co-clustering with the *DUX4*-positive cases. Interestingly, we found 12 identical, differentially expressed isoforms (of which three were significant (FDR < 0.05) and nine were not significant (FDR < 1)), with 100% concordance in the direction of regulation in the *DUX4* subgroup (Table S6). When we compared the differentially expressed isoforms of the *DUX4* subgroup with the other subgroups, there were, at most, four non-significant (FDR < 1) co-occurring isoforms in most cases regulated in the opposite directions. This specific correspondence between the differential isoform profiles of the *DUX4*-positive cases and B-other cases co-clustering with *DUX4*-positive cases provides additional leverage in the resolution of the unclassified B-other cases. The complete differential splicing analysis output is presented in File S3.

### 3.3. Mutation Analysis of Whole-Transcriptome BCP-ALL Data Has Limited Applicability Due to High Level of RNA Editing Events

**Figure 2:**
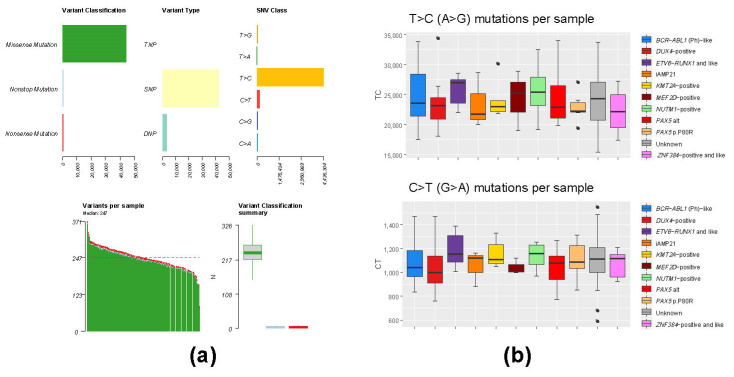
Mutation analysis of the entire cohort and the classified BCP-ALL subgroups does not show subgroup-specific deviation of mutation rates. The overall mutation rate statistics for the entire cohort are shown in (**a**). Median distribution of the same statistics within BCP-ALL subgroups does not show significant deviation in the number of T > C (A > G) substitutions or C > T (G > A) substitutions (**b**).

In addition to structural rearrangements, previous studies have also identified recurrent and mutually exclusive mutations in the group of B-other cases, e.g., *PAX5* p.P80R and *IKZF1* p.N159Y, for which it was shown to drive the development of ALL [34,49,50]. Furthermore, various studies have shown that somatic mutations associated with treatment resistance and the development of relapse can already be present at diagnosis [26,51,52]. In order to identify patients with ALL driving mutations in *PAX5* and *IKZF1*, as well as to examine the applicability of the whole-transcriptome data to identify relapse-driving mutations in ALL, we performed mutation analysis. To our surprise, we observed a large number of T > C substitutions in all the samples included in this cohort (Figure 2). The relative abundance of T > C substitutions is consistent with the presence of a previously described post-transcriptional RNA editing of adenine to inosine, resulting in mainly T > C and A > G substitutions [53,54]. 

Despite these RNA editing events, our analysis unraveled six cases in total with *PAX5* p.P80R mutation, as well as one case with *IKZF1* p.N159Y. In addition to leukemia-driving mutations, we have identified recurrent pathogenic mutations *CRLF2* p.F232C (*n* = 7), *TYK2* p.A413T (*n* = 7), *JAK2* p.R683G (*n* = 4), *KRAS* p.G12D (*n* = 4), *FLT3* p.Y842C (*n* = 3) and *PTPN11* p.D61V (*n* = 3), previously frequently found in pediatric ALL (Table S4 and Figure S1). 

### 3.4. EDTA Tubes Are a Viable Alternative to PAXgene RNA Stabilizing Tubes

Extraction of the high-quality RNA is pivotal for the reliable detection of fusion transcripts and differential expression analysis. However, obtaining high-quality RNA can be hampered by storage and shipping conditions of the biological material to diagnostic laboratories. Although the introduction of sampling tubes containing RNA stabilizing agents may be beneficial when RNA cannot be extracted in a short time after sampling, the use of different tubes for the samples that will be analyzed with different techniques is logistically complex and connected with higher expenses. In order to examine the suitability of commonly used EDTA and PAXgene RNA stabilizing tubes for extracting high-quality RNA and analysis of whole transcriptomes, we collected 12 bone marrow samples from six children. Two matched samples for each child were obtained from the same bone marrow puncture and stored in EDTA and PAXgene RNA stabilizing tubes. We subsequently performed RNA extraction (<24 h after BM puncture), confirmed the quality of RNA and performed RNA sequencing in order to investigate the differences in the detected fusion transcripts, mutations and differentially expressed genes of RNA obtained from material stored in EDTA or PAXgene RNA stabilizing tubes. 

#### 3.4.1. Comparison of Sequencing Output (PAXgene vs. EDTA) Showed Slightly Higher Duplication Rate in PAXgene Samples

Using FastQC [55] and Picard-tools [56], we compared sequencing characteristics of the samples stored in PAXgene RNA stabilizing tubes and EDTA tubes. We did not observe major variations in sequencing characteristics between the samples. In addition, as measured by the MarkDuplicates tool of Picard-tools, the mean duplication rate of samples derived from PAXgene RNA stabilizing tubes was 37.68% (26.03–51.38%), while the mean duplication rate of samples derived from EDTA tubes was 34.54% (24.40–43.31%). However, this difference is not statistically significant (*p*-val = 0.70, Mann–Whitney U test). Of note, these high duplication rates do not only represent PCR duplicates but also natural duplication due to high sequencing coverage associated with gene expression. 

#### 3.4.2. Influence of the Different Storage Tubes on the Overall Gene Expression

**Figure 3:**
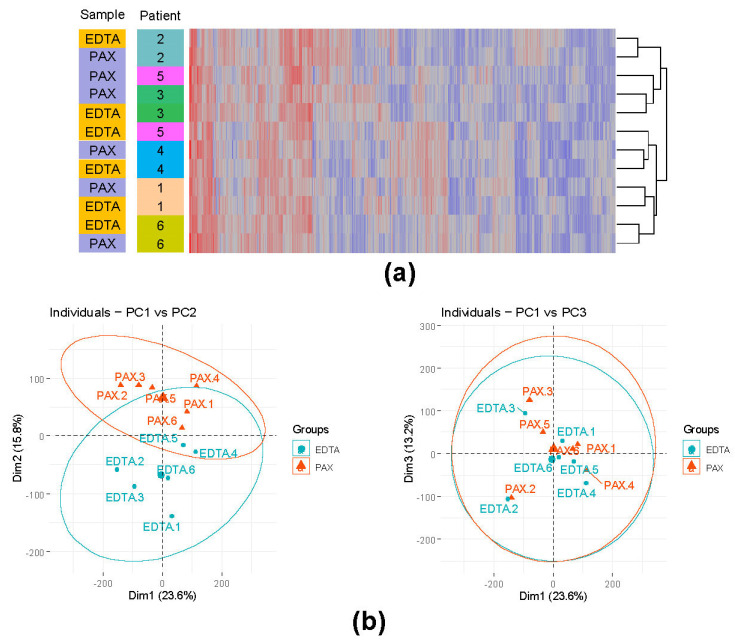
PAXgene RNA stabilized samples and EDTA samples of the same individuals vary in gene expression and mutation (RNA editing) rates. A heatmap of the top 1000 most variable genes and hierarchical clustering revealed that, despite the differences in the gene expression between PAXgene RNA stabilizing tubes and EDTA tubes, samples from the same individual cluster together (**a**). This was further reinforced with the principal component analysis (**b**), with the second component reflecting the gene expression variability caused by the sample storage tubes.

Previous studies showed that the lack of RNA stabilization can result in expression bias for particular transcripts in the EDTA tubes [57]. Therefore, we conducted differential gene expression analysis customized for paired data (see Methods). Only genes with an average read count >10 were considered. Without filtration based on the fold change (PAXgene vs. EDTA), we found 2626 upregulated and 2643 downregulated DEGs. We further identified three groups of DEGs based on the effect size, showing DEGs progressively more affected by the tube technology. In group 1 with the fold change cutoff (Log2(fold change) >1, we had 4135 DEGs (2075 upregulated and 2060 downregulated). In group 2 with the fold change cutoff (Log2(fold change) >2, we had 1202 DEGs (607 upregulated and 595 downregulated). In group 3 with the fold change cutoff (Log2(fold change) >3, we had 310 DEGs (143 upregulated and 167 downregulated). We further performed hierarchical clustering and principal component analysis (PCA) on expression data of the samples derived from PAXgene RNA stabilizing tubes and EDTA tubes (Figure 3a,b). The expression data included all genes with at least 10 mapped reads in every sample. Hierarchical clustering revealed that the majority of the samples derived from the same individual cluster together. Interestingly, PCA analysis revealed that the groups are primarily separated by the second component, explaining 15.8% of variance. Conversely, the first component (23.6%) and third component (13.2%) had lower influence on sample separation by the RNA stabilization technology and likely represent biological characteristics of the samples. These findings suggest that the use of different sample storage tubes needs to be carefully considered in the applications that require differential expression analysis. Although PCA analysis and hierarchical clustering demonstrated high similarity between samples from the same individual stored in the PAXgene RNA stabilizing tubes or EDTA tubes, diagnostic laboratories should abstain from using different sample storage tubes in order to avoid batch effects. 

#### 3.4.3. No Appreciable Correlation between Expression and Decay Constants Exists in Either EDTA or PAXgene Stored Samples

We next tested the correlation of TPM expression values and decay constants of the DEGs in both PAXgene and EDTA samples. We used the decay constants published by Romero et al. [58]. The corresponding scatter plots with regression lines are shown in Figure S2. Interestingly, as shown in Figure S3, upregulated DEGs (PAXgene vs. EDTA), i.e., the downregulated DEGs in the EDTA vs. PAXgene, have somewhat lower decay rates, suggesting that genes over-abundant in EDTA samples are not dominated by slow-degrading transcripts. Therefore, gene expression analysis appears to be unaffected by the expected higher rate of RNA degradation in EDTA samples. 

#### 3.4.4. Fusion Detection Assessment (PAXgene vs. EDTA) 

Next, we examined if the use of EDTA or PAXgene RNA stabilizing tubes may hamper our ability to accurately detect the presence of fusion transcripts. Of note, read coverage was not significantly different in the PAX vs. EDTA samples (median ± SD: 117.7 ± 6.7 million read pairs in PAXgene vs. 118.7 ± 17.7 million read pairs in EDTA). Using karyotyping and FISH, we detected *ETV6-RUNX1* fusion in two cases, *IGH-CRLF2* in one, while in the remaining three, no relevant B-ALL stratifying alterations were identified (B-other subgroup). Using whole-transcriptome data, we detected *ETV6-RUNX1* fusions in both EDTA samples, while this fusion was only detected in one of the corresponding PAXgene samples. The PAXgene sample where we failed to detect this fusion had 115.3 million read pairs compared to 120.9 million read pairs in the corresponding EDTA sample. The *IGH-CRLF2* fusion was not detected in either of the corresponding samples by RNA sequencing, which is in line with previous studies, indicating that the fusions involving non-transcribed *IGH* loci are challenging to detect [29,59]. 

### 3.5. Low Blast Count Samples Can Be Reliably Profiled by Whole-Transcriptome RNA-seq Given Sufficient Sequencing Depth

The major issue in the routine diagnostics are samples with low numbers of leukemic blasts, which can hamper the reliable detection of fusion transcripts. In order to determine the ability of our analysis pipeline to detect gene fusions present in a limited number of leukemic blasts, we analyzed five samples with known fusions. These included three *BCR-ABL1*-positive samples (blast count: 33%, 20% and 7%; read coverage in millions of read pairs: 89.4, 113.6 and 88.4) and two *ETV6-RUNX1*-positive samples (blast count 20% and 7.5%; read coverage in millions of read pairs: 94.3 and 102.7). In all cases, our fusion pipeline reliably detected the fusions. 

## 4. Discussion

BCP-ALL is a genetically heterogeneous disease, in which different alterations show strong associations with the treatment outcome [9,11,14,25,26,51,52]. Therefore, improvements in genetic characterization of the BCP-ALL are relevant for risk stratification of patients with genetic compositions associated with unfavorable prognoses, as well as the identification of new and potentially targetable genetic alterations. The detection of rare genomic alterations is frequently limited by the approaches currently used in the routine diagnostics, and the introduction of high-throughput, genome-wide next generation sequencing assays in the routine diagnostic can improve the detection of these rare alterations and aid risk-based treatment stratification. In this study, we examined the applicability of the whole-transcriptome analysis for the routine diagnostics and validated our findings using independent approaches. We specifically focus on the B-other ALL cases, in which relevant risk stratification markers cannot be detected using conventional cytogenetic methods. The most obvious example of previously unassigned B-other samples clustering with a known BCP-ALL subgroup are the unknown samples in the *DUX4*-positive cluster. Differential splicing analysis showed a high degree of overlap in differential isoform expression between *DUX4*-positive and the co-clustering B-other samples, highlighting the utility of differential splicing analysis in addition to expression clustering in diagnostics and personalized patient care. Previous studies have shown that the presence of *ERG* deletions, an event characterizing *DUX4*-positive ALL, neutralizes the otherwise adverse prognostic effect of *IKZF1* deletions [60,61]. In combination with other genes involved in the B-cell development (IKZF1plus signature), the presence of *IKZF1* deletions represents a marker currently used for risk stratification in different treatment protocols, including AIEOP-BFM ALL 2017 [62]. It remains to be explored whether in *DUX4*-positive ALL, the IKZF1plus signature maintains its association with a poor prognosis, and, if this is not the case, whether unnecessary treatment intensification can be avoided. 

In this study, we demonstrated that the detection of relevant risk-stratifying genomic alterations, i.e., *ETV6-RUNX1* and *BCR-ABL1*, could be reliably performed even in the samples with a low blast percentage. However, the detection of lowly expressed fusion transcripts may be more challenging, particularly in the samples with very low blast percentages. Furthermore, the diminishment of unique expression patterns by a low number of blasts may render classification impossible in cases without discerning genomic alterations, e.g., *ETV6-RUNX1*-like and *BCR-ABL1*-like.

As reported in the Results section, the detected variants in whole-transcriptome samples are dominated by RNA editing events and, therefore, have limited value. Nevertheless, mutation analysis in our study still identified the key diagnostic mutations, such as *PAX5* p.P80R and *IKZF1* p.N159Y. 

While RNA stabilization qualities of the PAXgene RNA stabilizing tubes are likely to prolong sample viability and minimize effects of long-term storage on RNA expression [57], their use is connected with higher costs and is logistically complex. Therefore, in this study, we analyzed the applicability of commonly used EDTA tubes for short-term sample storage (<24 h) prior to RNA extraction. Our PCA analysis and hierarchical clustering revealed high similarity between samples obtained from individual patients. Furthermore, we were able to confirm all expected fusion transcripts, suggesting that EDTA tubes are a viable alternative to the PAXgene RNA stabilizing tubes for short-term sample storage. This finding is particularly relevant for hospitals and diagnostic laboratories since the use of broadly available EDTA tubes reduces the costs and simplifies sample acquisition and sample processing. RNA isolation from PAXgene RNA stabilizing tubes is connected with a longer processing time, which represents important considerations in situations when the time to conduct the necessary diagnostic procedures is limited. Furthermore, samples taken using EDTA tubes can be processed for multiple techniques simultaneously, e.g., FISH, karyotyping and immunophenotyping, while viable mononuclear cells can be isolated and DMSO frozen for later use. Finally, the ability to perform the isolation of mononuclear cell fraction and subsequent enrichment for the tumor population are particularly important for samples with a low blast percentage due to the limited number of fusion transcripts and the dilution of expression patterns in the bulk bone marrow sample. On the other hand, when prolonged storage cannot be avoided, PAXgene RNA stabilizing tubes outperform EDTA tubes in terms of preserving RNA concentration and RNA yield in blood samples [57]. Therefore, consideration regarding the usage of PAXgene RNA stabilizing or EDTA tubes largely depends on the needs of diagnostic centers and speed in which samples can be processed. While the discoveries presented here may affect experimental and diagnostics routines, we have to concede that this sub-study is somewhat preliminary due to a relatively low cohort size (six patients). In summary, we can state that the use of EDTA tubes for sample collection instead of PAXgene RNA stabilizing tubes does not have adverse effects on sequencing and downstream analysis for diagnostic purposes.

## 5. Conclusions

Taken together, our data demonstrate the applicability and limitations of whole-transcriptome analysis for routine diagnostics and further refinement of cases without known risk stratification markers. This was particularly relevant for a large portion of ALL samples co-clustering together with *DUX4*-positive cases. Furthermore, we have demonstrated that the whole-transcriptome analysis can be successfully implemented, even in samples with a low blast percentage, and that the use of tubes with EDTA does not hamper the quality of the RNA and the whole-transcriptome data generated using this material. 

## Data Availability

The data presented in this study are available on request from the corresponding author.

## Supplementary Materials

The following are available online at https://www.mdpi.com/article/10.3390/cancers13225653/s1, Figure S1: Heatmap showing mutation status in the genes frequently mutated in the diagnosis samples of B-other patients included in the study. Figure S2: No appreciable correlation between expression (TPM) and decay constant exists in either EDTA or PAXgene samples, Figure S3: Slow degrading DEGs are not overrepresented in genes upregulated in the EDTA samples, Table S1: Clinical characteristics of patients included in the study, Table S2: Validation of detected fusions using karyotyping, FISH analysis on metaphase and interphase nuclei, arrayCGH analysis and immunophenotyping, Table S3: The “white list” of acute lymphocytic leukemia (ALL)-relevant genes obtained from the literature, Table S4: Pathogenic mutations by subgroup, Table S5: Detected fusions by BCP-ALL subgroup, Table S6: Differentially spliced isoforms in the *DUX4* subgroup and their counterparts in the other subgroups (including samples co-clustering with the *DUX4* subgroup based on gene expression), File S1: Output of the fusion detection programs, File S2: Differentially expressed genes between ALL subtypes, File S3: Differentially spliced genes per ALL subgroups.
