# Supplementary material for: Transcriptional and Mutational Profiling of B-Other Acute Lymphoblastic Leukemia for Improved Diagnostics
Source: Cancers (Basel). 2021 Nov 12;13(22):5653. doi: 10.3390/cancers13225653 (PMC8616234; doi:10.3390/cancers13225653)
Supplement: Supplementary file 1 [file cancers-13-05653-s001.zip › cancers-1434265-supplementary/supplementary figures_cancers-1434265_revised.pdf]

SUPPLEMENTARY FIGURES

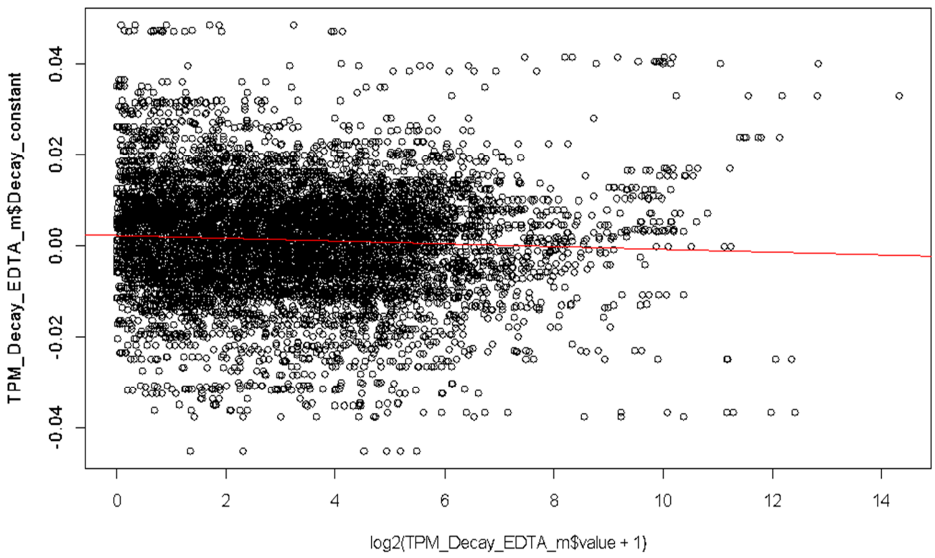

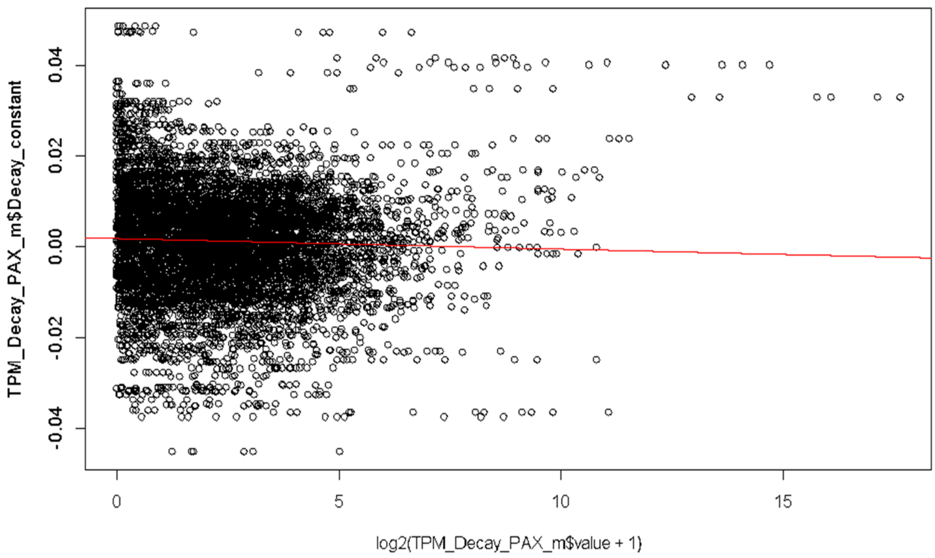

Figure S2. No appreciable correlation between expression (TPM) and decay constant exists in either EDTA or PAXgene samples.

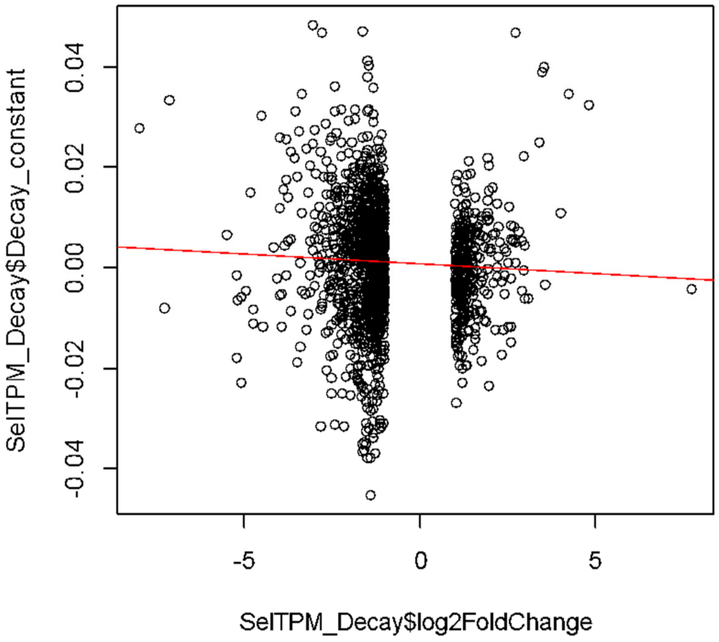

Figure S3. Slow degrading DEGs are not overrepresented in genes upregulated in the EDTA samples. On the left side are genes with higher relative abundance in EDTA. The gap shows exclusion of DEGs with |L2FC|<=1. On the right side are the genes with higher relative abundance in PAXgene. DEGs above 0 on the Y axis are degrading faster than the ones below 0. From this representation, we can reject the hypothesis that slow decaying DEGs dominate the upregulated genes in EDTA.
